# Supplementary material for: Trampolining Accidents in an Adult Emergency Department: Analysis of Trampolining Evolution Regarding Severity and Occurrence of Injuries
Source: Int J Environ Res Public Health. 2022 Jan 22;19(3):1212. doi: 10.3390/ijerph19031212 (PMC8834515; doi:10.3390/ijerph19031212)
Supplement: Supplementary file 1 [file ijerph-19-01212-s001.zip › ijerph-1529831-supplementary.pdf]

Supplementary

Table S1. Comparison between the age groups 16 to 30 and 31 to 63 years of age.

	Total		Age Group 31–62		Age Group 16–30		*p-value*
	N	%	*N*	%	*N*	%	
**Sex**							0.878
Male	88	61.1	15	62.5	73	60.8	
Female	56	38.9	9	37.5	47	39.2	
**Season of Consultation, n %**							0.172
Winter	42	29.2	3	12.5	39	32.5	
Spring	33	22.9	6	25.0	27	22.5	
Summer	30	20.8	8	33.3	22	18.3	
Autumn	39	27.1	7	29.2	32	26.7	
**Weekday of Consultation**							0.957
Monday	13	9.0	1	4.2	12	10.0	
Tuesday	19	13.2	4	16.7	15	12.5	
Wednesday	24	16.7	4	16.7	20	16.7	
Thursday	22	15.3	3	12.5	19	15.8	
Friday	20	13.9	3	12.5	17	14.2	
Saturday	32	22.2	6	25.0	26	21.7	
Sunday	14	9.7	3	12.5	11	9.2	
**Costs**							0.126
Up to 1000 CHF	58	40.3	9	37.5	49	40.8	
1001–10.000 CHF	42	29.2	10	41.7	32	26.7	
> 10.000 CHF	10	6.9	3	12.5	7	5.8	
No information	34	23.6	2	8.3	32	26.7	
**BMI kg/m^2**							**0.004***
Up to 18.5	4	2.8	3	12.5	1	0.8	
18.5–25	64	44.4	7	29.2	57	47.5	
> 25	22	15.3	6	25.0	16	13.3	
No information	54	37.5	8	33.3	46	38.3	
**Triage**							0.468
Acute life-threating	4	2.8	1	4.2	3	2.5	
High urgency	17	11.8	4	16.7	13	10.8	
Urgency	76	52.8	15	62.5	61	50.8	
Less urgency	10	6.9	1	4.2	9	7.5	
No information	37	25.7	3	12.5	34	28.3	
**Treatment Area**							0.133
Surgery	88	61.1	10	41.7	78	65.0	
Fasttrack	39	27.1	9	37.5	30	25.0	
Neurology	1	0.7	0	0.0	1	0.8	
Orthopaedic	6	4.2	2	8.3	4	3.3	
Ophthalmology	1	0.7	0	0.0	1	0.8	

Cranio-maxillo-facial surgery	3	2.1	0	0.0	3	2.5	
Ears-nose-throat	3	2.1	2	8.3	1	0.8	
Internal medicine	3	2.1	1	4.2	2	1.7	
**Route of Admission**							1.000
Self admission	84	58.3	14	58.3	70	58.3	
Other hospital	7	4.9	1	4.2	6	5.0	
General practitioner	6	4.2	1	4.2	5	4.2	
Ambulance	20	13.9	3	12.5	17	14.2	
Air-ambulance	5	3.5	1	4.2	4	3.3	
No information	22	15.3	4	16.7	18	15.0	
**Route of Discharge**							**0.006***
Home	118	81.9	18	75.0	100	83.3	
Hospitalised	24	16.7	4	16.7	20	16.7	
Transfer to a different hospital	2	1.4	2	8.3	0	0.0	
**Sites of Accident**							**0.005***
School sports lessons	24	16.7	1	4.2	23	19.2	
Indoor recreation hall	15	10.4	7	29.2	8	6.7	
Private	1	0.7	0	0.0	1	0.8	
No information	104	72.2	16	66.7	88	73.3	
**Mechanism of Injury**							0.833
Ankle sprain on the trampoline and next to trampoline	39	27.1	9	37.5	30	25.0	
Collision on the trampoline	23	16.0	4	16.7	19	15.8	
Collision next to trampoline	35	24.3	4	16.7	31	25.8	
Distortion	23	16.0	4	16.7	19	15.8	
Contusion by self	12	8.3	2	8.3	10	8.3	
Contusion with second person	3	2.1	1	4.2	2	1.7	
Additional object	1	0.7	0	0.0	1	0.8	
No acute trauma	2	1.4	0	0.0	2	1.7	
No information	6	4.2	0	0.0	6	5.0	
**Type of Injury**							0.414
Contusion	16	11.1	1	4.2	15	12.5	
Distortion	59	41.0	13	54.2	46	38.3	
Fracture	47	32.6	8	33.3	39	32.5	
Soft tissue injury	17	11.8	1	4.2	16	13.3	
Traumatic brain injury	5	3.5	1	4.2	4	3.3	
**Traumatic Brain Injury**							0.839
Yes	5	3.5	1	4.2	4	3.3	
No	139	96.5	23	95.8	116	96.7	
**Type of Trauma**							0.644
Combined without life-threatening injuries	9	6.2	2	8.3	7	5.8	
Monotrauma	135	93.8	22	91.7	113	94.2	

**Fracture**							0.937
Yes	49	34.0	8	33.3	41	34.2	
No	95	66.0	16	66.7	79	65.8	
**Region of Fracture**							
Upper extremities	7	4.9	0	0.0	7	5.8	0.363
Lower extremities	17	11.8	5	20.8	12	10.0	
Cervical spine	5	3.5	0	0.0	5	4.2	
Thoracic spine/ribs	4	2.8	0	0.0	4	3.3	
Lumbar spine	5	3.5	0	0.0	5	4.2	
Face, Head	10	6.9	3	12.5	7	5.8	
Clavicula	1	0.7	0	0.0	1	0.8	
No fracture	95	66.0	16	66.7	79	65.8	
**Region of Injury**							0.787
Upper extremities	11	7.6	1	4.2	10	8.3	
Lower extremities	69	47.9	14	58.3	55	45.8	
Cervical spine	21	14.6	4	16.7	17	14.2	
Thoracic spine/ribs	11	7.6	1	4.2	10	8.3	
Lumbar spine	8	5.6	0	0.0	8	6.7	
Face, Head	21	14.6	4	16.7	17	14.2	
Clavicula	1	0.7	0	0.0	1	0.8	
Multiple	2	1.4	0	0.0	2	1.7	
**Length of Stay in Hospital**							0.082
1 < 24h	120	83.3	17	70.8	103	85.8	
1–5	11	7.6	2	8.3	9	7.5	
> 5	13	9.0	5	20.8	8	6.7	

Table S2. Comparison between genders.

	**Total**		**Male**		**Female**		***p-value***
	N	%	*N*	%	*N*	%	
**Age Group**							0.606
16–25	98	68.1	41	73.2	57	64.8	
26–35	27	18.8	8	14.3	19	21.6	
36–45	10	6.9	3	5.4	7	8.0	
46–55	6	4.2	2	3.6	4	4.5	
56–65	3	2.1	2	3.6	1	1.1	
**Season of Consultation**							0.543
Winter	42	29.2	16	28.6	26	29.5	
Spring	33	22.9	11	19.6	22	25.0	
Summer	30	20.8	15	26.8	15	17.0	
Autumn	39	27.1	14	25.0	25	28.4	
**Weekday of Consultation**							0.916
Monday	13	9.0	5	8.9	8	9.1	
Tuesday	19	13.2	6	10.7	13	14.8	

Wednesday	24	16.7	10	17.9	14	15.9	
Thursday	22	15.3	7	12.5	15	17.0	
Friday	20	13.9	9	16.1	11	12.5	
Saturday	32	22.2	12	21.4	20	22.7	
Sunday	14	9.7	7	12.5	7	8.0	
**Costs**							0.258
Up to 1000 CHF	58	40.3	25	44.6	33	37.5	
1001–10.000 CHF	42	29.2	16	28.6	26	29.5	
> 10.000 CHF	10	6.9	1	1.8	9	10.2	
No information	34	23.6	14	25.0	20	22.7	
**BMI kg/m^2**							0.068
Up to 18.5	4	2.8	3	5.4	1	1.1	
18.5–25	64	44.4	22	39.3	42	47.7	
> 25	22	15.3	5	8.9	17	19.3	
No information	54	37.5	26	46.4	28	31.8	
**Triage**							0.068
Acute life-threating	4	2.8	4	7.1	0	0.0	
High urgency	17	11.8	8	14.3	9	10.2	
Urgency	76	52.8	28	50.0	48	54.5	
Less urgency	10	6.9	5	8.9	5	5.7	
No information	37	25.7	11	19.6	26	29.5	
**Treatment Area**							0.240
Surgery	88	61.1	36	64.3	52	59.1	
Fasttrack	39	27.1	16	28.6	23	26.1	
Neurology	1	0.7	1	1.8	0	0.0	
Orthopaedic	6	4.2	2	3.6	4	4.5	
Ophthalmology	1	0.7	1	1.8	0	0.0	
Cranio-maxillo-facial surgery	3	2.1	0	0.0	3	3.4	
Ears-nose-throat	3	2.1	0	0.0	3	3.4	
Internal medicine	3	2.1	0	0.0	3	3.4	
**Route of Admission**							**0.011***
Self admission	84	58.3	32	57.1	52	59.1	
Other hospital	7	4.9	0	0.0	7	8.0	
General practitioner	6	4.2	4	7.1	2	2.3	
Ambulance	20	13.9	8	14.3	12	13.6	
Air-ambulance	5	3.5	5	8.9	0	0.0	
No information	22	15.3	7	12.5	15	17.0	
**Route of Discharge**							0.439
Home	118	81.9	43	76.8	75	85.2	
Hospitalised	24	16.7	12	21.4	12	13.6	
Transfer to a different hospital	2	1.4	1	1.8	1	1.1	
**Sites of Accident**							0.625
School sports lessons	24	16.7	10	17.9	14	15.9	
Indoor recreation hall	15	10.4	4	7.1	11	12.5	

Private	1	0.7	0	0.0	1	1.1	
No information	104	72.2	42	75.0	62	70.5	
**Mechanism of Injury**							
Ankle sprain on and next to the trampoline	39	27.1	10	17.9	29	33.0	0.065
Collision on the trampoline	23	16.0	10	17.9	13	14.8	
Collision next to trampoline	35	24.3	15	26.8	20	22.7	
Distortion	23	16.0	14	25.0	9	10.2	
Contusion by self	12	8.3	3	5.4	9	10.2	
Contusion with second person	3	2.1	0	0.0	3	3.4	
Additional object	1	0.7	0	0.0	1	1.1	
No acute trauma	2	1.4	0	0.0	2	2.3	
No information	6	4.2	4	7.1	2	2.3	
**Type of Injury**							
Contusion	16	11.1	5	8.9	11	12.5	0.736
Distortion	59	41.0	21	37.5	38	43.2	
Fracture	47	32.6	22	39.3	25	28.4	
Soft tissue injury	17	11.8	6	10.7	11	12.5	
Traumatic brain injury	5	3.5	2	3.6	3	3.4	
**Traumatic Brain Injury**							
Yes	5	3.5	2	3.6	3	3.4	0.959
No	139	96.5	54	96.4	85	96.6	
**Type of Trauma**							
Combined without life-threatening injuries	9	6.2	6	10.7	3	3.4	0.077
Monotrauma	135	93.8	50	89.3	85	96.6	
**Fracture**							
Yes	49	34.0	23	41.1	26	29.5	0.155
No	95	66.0	33	58.9	62	70.5	
**Region of Fracture**							
Upper extremities	7	4.9	3	5.4	4	4.5	0.110
Lower extremities	17	11.8	9	16.1	8	9.1	
Cervical spine	5	3.5	4	7.1	1	1.1	
Thoracic spine/ribs	4	2.8	3	5.4	1	1.1	
Lumbar spine	5	3.5	1	1.8	4	4.5	
Face, Head	10	6.9	2	3.6	8	9.1	
Clavicula	1	0.7	1	1.8	0	0.0	
No fracture	95	66.0	33	58.9	62	70.5	
**Region of Injury**							
Upper extremities	11	7.6	4	7.1	7	8.0	0.149
Lower extremities	69	47.9	27	48.2	42	47.7	
Cervical spine	21	14.6	13	23.2	8	9.1	
Thoracic spine/ribs	11	7.6	4	7.1	7	8.0	
Lumbar spine	8	5.6	1	1.8	7	8.0	

Face, Head	21	14.6	5	8.9	16	18.2	
Clavicula	1	0.7	1	1.8	0	0	
No fracture	2	1.4	1	1.8	1	1.1	
**Length of Stay in Hospital**							
1 < 24h	120	83.3	51	91.1	69	78.4	
1–5	11	7.6	2	3.6	9	10.2	0.135
> 5	13	9.0	3	5.4	10	11.4	

Table S3. Comparison between two time periods.

	**Total**		**Year of Consultation 2012-2020**		**Year of Consultation 2003-2011**		***p-value***
	N	%	*N*	%	*N*	%	
**Age Group**							0.258
16–25	98	68.1	75	67.6	23	69.7	
26–35	27	18.8	18	16.2	9	27.3	
36–45	10	6.9	9	8.1	1	3.0	
46–55	6	4.2	6	5.4	0	0.0	
56–65	3	2.1	3	2.7	0	0.0	
**Sex**							0.946
Male	88	61.1	68	61.1	20	60.6	
Female	56	38.9	43	38.7	13	39.4	
**Season of Consultation**							0.768
Winter	42	29.2	31	27.9	11	33.3	
Spring	33	22.9	26	23.4	7	21.2	
Summer	30	20.8	25	22.5	5	15.2	
Autumn	39	27.1	29	26.1	10	30.3	
**Weekday of Consultation**							0.134
Monday	13	9.0	9	8.1	4	12.1	
Tuesday	19	13.2	18	16.2	1	3.0	
Wednesday	24	16.7	15	13.5	9	27.3	
Thursday	22	15.3	16	14.4	6	18.2	
Friday	20	13.9	14	12.6	6	18.2	
Saturday	32	22.2	26	23.4	6	18.2	
Sunday	14	9.7	13	11.7	1	3.0	
**Costs**							**<0.001***
Up to 1000 CHF	58	40.3	58	52.3	0	0.0	
1001–10.000 CHF	42	29.2	42	37.8	0	0.0	
> 10.000 CHF	10	6.9	10	9.0	0	0.0	
No information	34	23.6	1	0.9	33	100.0	
**BMI kg/m^2**							**<0.001***
Up to 18.5	4	2.8	4	3.6	0	0.0	
18.5–25	64	44.4	64	57.7	0	0.0	
> 25	22	15.3	18	16.2	4	12.1	

No information	54	37.5	25	22.5	29	87.9	
**Triage**							**<0.001***
Acute life-threating	4	2.8	3	2.7	1	3.0	
High urgency	17	11.8	15	13.5	2	6.1	
Urgncy	76	52.8	71	64.0	5	15.2	
Less urgency	10	6.9	6	5.4	4	12.1	
No information	37	25.7	16	14.4	21	63.6	
**Treatment Area**							**0.047***
Surgery	88	61.1	63	56.8	25	75.8	
Fasttrack	39	27.1	34	30.6	5	15.2	
Neurology	1	0.7	0	0.0	1	3.0	
Orthopaedic	6	4.2	6	5.4	0	0.0	
Ophthalmology	1	0.7	1	0.9	0	0.0	
Cranio-maxillo-facial surgery	3	2.1	1	0.9	2	6.1	
Ears-nose-throat	3	2.1	3	2.7	0	0.0	
Internal medicine	3	2.1	3	2.7	0	0.0	
**Route of Admission**							0.196
Self admission	84	58.3	62	55.9	22	66.7	
Other hospital	7	4.9	4	3.6	3	9.1	
General practitioner	6	4.2	5	4.5	1	3.0	
Ambulance	20	13.9	16	14.4	4	12.1	
Air-ambulance	5	3.5	3	2.7	2	6.1	
No information	22	15.3	21	18.9	1	3.0	
**Route of Discharge**							**0.046***
Home	118	81.9	95	85.6	23	69.7	
Hospitalised	24	16.7	14	12.6	10	30.3	
Transfer to a different hospital	2	1.4	2	1.8	0	0.0	
**Sites of Accident**							0.260
School sports lessons	24	16.7	16	14.4	8	24.2	
Indoor recreation hall	15	10.4	14	12.6	1	3.0	
Private	1	0.7	1	0.9	0	0.0	
No information	104	72.2	80	72.1	24	72.7	
**Mechanism of Injury**							0.218
Ankle sprain on the trampoline and next to trampoline	39	27.1	27	24.3	12	36.4	
Collision on the trampoline	23	16.0	21	18.9	2	6.1	
Collision next to trampoline	35	24.3	23	20.7	12	36.4	
Distortion	23	16.0	20	18.0	3	9.1	
Contusion by self	12	8.3	11	9.9	1	3.0	

Contusion with second person	3	2.1	2	1.8	1	3.0	
Additional object	1	0.7	1	0.9	0	0.0	
No acute trauma	2	1.4	2	1.8	0	0.0	
No information	6	4.2	4	3.6	2	6.1	
**Type of Injury**							0.399
Contusion	16	11.1	14	13.5	2	5.0	
Distortion	59	41.0	42	40.4	17	42.5	
Fracture	47	32.6	31	29.8	16	40.0	
Soft tissue injury	17	11.8	14	13.5	3	7.5	
Traumatic brain injury	5	3.5	4	2.9	2	5.0	
**Traumatic Brain Injury**							0.535
Yes	5	3.5	3	2.9	2	5.0	
No	139	96.5	101	97.1	38	95.0	
**Type of Trauma**							0.443
Combined without life-threatening injuries	9	6.2	6	5.4	3	9.1	
Monotrauma	135	93.8	105	94.6	30	90.9	
**Fracture**							0.246
Yes	49	34.0	35	31.5	14	42.4	
No	95	66.0	76	68.5	19	57.6	
**Region of Fracture**							0.378
Upper extremities	7	4.9	5	4.5	2	6.1	
Lower extremities	17	11.8	11	9.9	6	18.2	
Cervical spine	5	3.5	3	2.7	2	6.1	
Thoracic spine/ribs	4	2.8	4	3.6	0	0.0	
Lumbar spine	5	3.5	4	3.6	1	3.0	
Face, Head	10	6.9	8	7.2	2	6.1	
Clavicula	1	0.7	0	0.0	1	3.0	
No fracture	95	66.0	76	68.5	19	57.6	
**Region of Injury**							0.408
Upper extremities	11	7.6	9	8.1	2	6.1	
Lower extremities	69	47.9	50	45.0	19	57.6	
Cervical spine	21	14.6	17	15.3	4	12.1	
Thoracic spine/ribs	11	7.6	10	9.0	1	3.0	
Lumbar spine	8	5.6	7	6.3	1	3.0	
Face, Head	21	14.6	17	15.3	4	12.1	
Clavicula	1	0.7	0	0.0	1	3.0	
No fracture	2	1.4	1	0.9	1	3.0	
**Length of Stay in Hospital**							0.739
1 < 24h	120	83.3	93	83.8	27	81.8	
1–5	11	9	9	8.1	2	6.1	
> 5	13	9	9	8.1	4	12.1	
